# Genetic Evaluation of Schizophrenia Using the Illumina HumanExome Chip

**DOI:** 10.1371/journal.pone.0150464

**Published:** 2016-03-30

**Authors:** Tim Moons, Marc De Hert, Edith Gellens, Leen Gielen, Kim Sweers, Sigrun Jacqmaert, Ruud van Winkel, Philippe Vandekerckhove, Stephan Claes

**Affiliations:** 1 GRASP research group, UPC KULeuven, Campus Leuven, Leuven, Belgium; 2 UPC KULeuven, campus Kortenberg, Kortenberg, Belgium; 3 Center for Human Genetics, Leuven, Belgium; 4 Belgian Red Cross-Flanders, Mechelen, Belgium; 5 KU Leuven—University of Leuven, Department of Public Health and Primary Care, Leuven, Belgium; Department of Psychiatry, CANADA

## Abstract

**Introduction:**

Schizophrenia is a genetically heterogeneous disorder that is associated with several common and rare genetic variants. As technology involved, cost advantages of chip based genotyping was combined with information about rare variants, resulting in the Infinium HumanExome Beadchip. Using this chip, a sample of 493 patients with schizophrenia or schizoaffective disorder and 484 healthy controls was genotyped.

**Results:**

From the initial 242901 SNVs, 88306 had at least one minor allele and passed quality control. No variant reached genomewide-significant results (p<10^-8^). The SNP with the lowest p-value was rs1230345 in WISP3 (p = 3.05*10^−6^), followed by rs9311525 in CACNA2D3 (p = 1.03*10^−5^) and rs1558557 (p = 3.85*10^−05^) on chromosome 7. At the gene level, 3 genes were of interest: WISP3, on chromosome 6q21, a signally protein from the extracellular matrix. A second candidate gene is CACNA2D3, a regulator of the intracerebral calcium pathway. A third gene is TNFSF10, associated with p53 mediated apoptosis.

## Introduction

Schizophrenia is a psychiatric disorder characterized by the presence of psychotic and negative symptoms and has a heterogeneous presentation and prognosis. Combined with schizoaffective disorder, it has an estimated lifetime prevalence of approximately 1%.[1,2] Schizophrenia has a high heritability (estimated between 65 and 81%),[3,4] and evidence suggests a polygenic inheritance, with an established role of both rare variants with large effects, as well as common Single Nucleotide Polymorphisms (SNPs) with small effects.[5,6] Given this complexity, early genetic studies failed to replicate previous associations, leading to a pessimistic outlook on schizophrenia genetics.[7]

Technological advances such as chip-based genotyping made large-scale studies using genome-wide information affordable and technically possible. Genome-wide association studies (GWAS) use tagging SNPs to identify common risks alleles, based upon the principle of linkage disequilibrium. Thus, by using only between 250 000 and 1 million SNPs, the whole genome is scanned for risk loci.

Although initial GWAS studies had limited (less than 500 cases) sample sizes and power,[8] subsequent studies with increasing sample sizes led to several common SNPs associated with schizophrenia.[9–22] Due to the nature of GWAS studies, incorporating many common variants, a stringent correction for multiple testing has to be applied (typically, Bonferroni correction with genome-wide significance defined as a p-value below 10^−8^), as well as independent replications.[23] The most recent study by the Psychiatric Genomics Consortium found 108 loci that obtained sufficiently low p-values to be associated with schizophrenia.[22]

A second technology that contributed to the knowledge of the genetic architecture of schizophrenia was next generation sequencing, which enables the identification of rare variants with minor allele frequencies below 5%. Sequencing allows for SNP genotyping, as well as for the detection of copy number variants,[24] but is expensive, slower than chip genotyping and requires additional techniques for data analysis. An increased burden of rare mutations has been found in schizophrenia.[25] Due to the high cost associated with sequencing studies, several studies were limited to either specific target regions,[26,27] or whole exome sequencing.[25,28–32]

The Human Exome consortium, incorporating researchers from different research domains such as schizophrenia and autism genetics,[33] jointly developed a SNP chip incorporating >240 000 putatively functional variants within the human exome. This chip was then marketed by Illumina, as the HumanExome Beadchip.[34] This chip was designed to be efficient towards genotyping cost and analysis burden, yet incorporating a large number of rare SNPs without adding the need for sequencing.

The current study used the HumanExome beadchip to detect rare variants in a sample of 484 patients with schizophrenia or schizoaffective disorder and 493 healthy volunteers recruited from the general population.

## Methods

### Sample

The current investigation uses samples from three sources: two different patient sets were used, and one set of controls. The patient sample consisted of 650 patients with psychotic spectrum disorder. Part of this sample was previously used for pharmacogenetic research [35–40]. Initial inclusion in this sample was based upon clinician diagnosis, and diagnosis was confirmed using the OPCRIT v4 questionnaire before inclusion in the current study.[41] These patients come from five different hospitals in Belgium (UPC St. Jozef, Kortenberg; Psychosociaal centrum St. Alexius, Elsene; UPC St. Kamillus, Bierbeek; Broeders Alexianen, Tienen and St. Amedeus, Mortsel).

A healthy control sample of both mentally and physically healthy plasma donors of Caucasian descent. They have never had any mental illness, have not been treated for mental illness and have never taken medication for mental disorders. This sample was obtained in collaboration with the Belgian Red Cross Flanders.

All patients and healthy controls gave written informed consent for genetic testing. After obtaining approval by the “Commissie medische ethiek” of the UZ Leuven, Leuven, Belgium, the study was approved by the local ethics committees of the coordinating and sampling hospitals and the Red Cross Belgium. The study was conducted in accordance with the current revision of the Helsinki declaration [42].

### DNA analysis

DNA was extracted from peripheral blood lymphocytes using a Chemagen MSMI (Perkin Elmer–Chemagen). Samples were genotyped using the Illumina HumanExome v1.1 chip. Extraction, storage and analysis was conducted in the Center for Human Genetics in Leuven, Belgium.

DNA quality control was done following the manufacturers' guidelines using GenomeStudio software (v2010.3).[43] Genotypes were called using the supplied cluster file, with automatic re-clustering of all genotypes with a call rate below 100%. After this re-clustering, all remaining genotypes with call rates below 100% were manually verified. All samples with exactly 12 rare allele homozygote or 12 heterozygote cases (12 equals the number of samples per chip) were manually checked to exclude chip effects. All SNPs on the X and Y chromosome were manually verified. Genotypes were automatically clustered using the OPTICALL software, and Single Nucleotide Variants (SNV, both SNP and indels or deletions) with major differences in call rates between both methods were manually verified and excluded when after manual verification, no consensus calling was obtained.[44]

Further quality control was done using PLINK v1.07.[45] Ethnicity and relatedness was verified using the MDS algorithm in the KING software v1.4, as with the build-in functions of PLINK.[46] Based upon the eigenvalues, the 3 first principal components were retained. The 08/2010 release of the 1000-genomes project was used to check population membership, and samples of non-Caucasian descent were excluded.

Analysis of autosomal SNPs was done using logistic regression with the first 3 principal components (PCA) generated by KING as covariates. A combined analysis of rare (MAF<0.03) and common variants was done using the CommonRare function implemented in SKAT version 0.91.[47] A multilevel logistic regression, using sex and the first three principal components generated by KING as covariates was used to assess associations on the X-chromosome.

## Results

### Sample descriptive

A sample of 1023 volunteers consisting of 525 cases with DSM-IV schizophrenia or schizoaffective disorder and 496 healthy controls was genotyped.

After exclusion of samples with call rate below 98% (n = 2), duplicate samples (n = 6), samples related up to the second degree (n = 11), sex errors (n = 6), samples with excess heterozygosity (n = 2) and samples of non-Caucasian descent or other problems (n = 29), a total of 977 samples consisting of 493 cases and 484 controls remained. S1 Fig plots the ethnicity of the current sample compared with the 1000 genomes database. An overview of the first and second principal component of the MDS algorithm is given in S2 Fig.

There was no significant difference in mean age between patients and controls (resp.44.8 vs. 44, t = -1.1341, p = .26), but there were significantly more males amongst the patients than controls (resp. 70.2 vs. 57.9% male, χ^2^ = 15.6, p = 7.82*10^−05^).

### DNA quality control

From the initial 242901 markers, a total of 242,401 SNVs (99.8%) passed all quality control measures. 129,453 (53.3%) SNVs were re-clustered using the built-in tool of GenomeStudio, resulting in 159,523 (65.7%) SNVs with 0 missing SNVs. The remaining 83,378 SNVs were manually verified. After this stage, QC according to Illumina’s guidelines was applied. In a final stage all SNVs on the X-chromosome were manually verified. In this QC phase, 282 SNPs were excluded from further analysis.

Using PLINK, we excluded 401 SNVs with > 2% missing alleles, 84 SNPs due to Hardy-Weinberg deviation (p < .0005), with 242,416 (99.8%) SNVs remaining for further analysis. Of these, 88,306 (36.4%) had at least one minor allele in one or more participants and were used in the subsequent analysis. S1 Data contains the cleaned PLINK data files after quality control.

### Single SNP analysis

**Fig 1:**
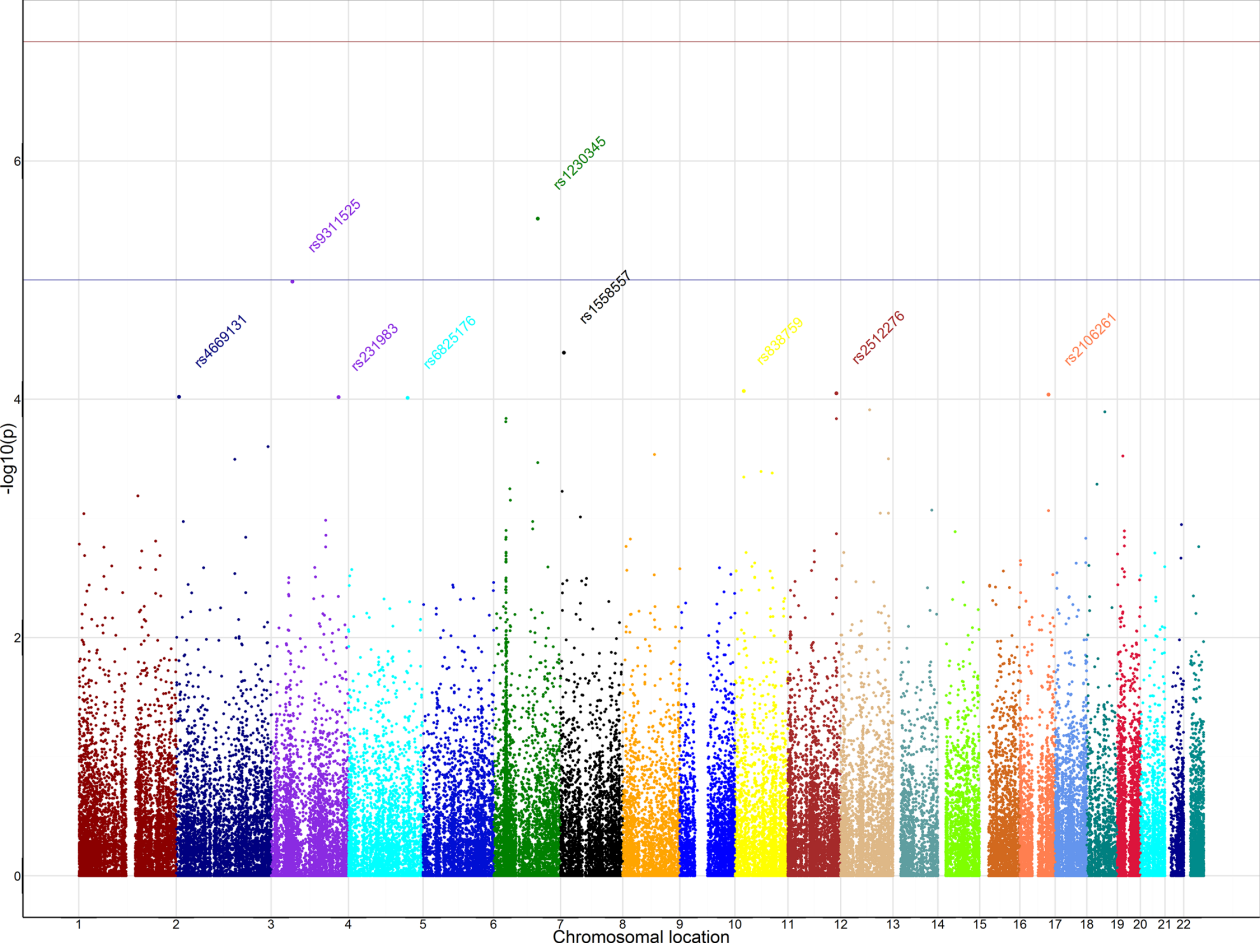
Manhattan plot of the autosomal chromosomes after logistic regression corrected for differences in ethnicity. Names of the top 9 SNPs were included in the plot.

No SNP reached genomewide significance (all p> 10^−8^). Three SNPs had p values lower than 5*10^−5^ in the corrected logistic regression. Table 1 lists all SNPs that obtained a p-value below 10^−4^. Fig 1 shows the Manhattan plot of the logistic regression. The QQ plot of the PCA corrected logistic regression is shown in S3 Fig.

**Table 1:** Autosomal SNPs that had a p-value below 10^−4^ after logistic regression using the first 3 principal components.

SNP	CHR	BP	n	OR	β	P	major/minor	Freq_Cases_	Freq_Controls_	Gene
rs1230345	6	112382313	977	1.639	4.668	3.05E-06	C/A	0.330	0.237	WISP3
rs9311525	3	54183550	976	0.663	-4.411	1.03E-05	G/A	0.372	0.470	CACNA2D3
rs1558557	7	8308993	977	0.688	-4.103	4.08E-05	G/A	0.394	0.489	
rs838759	10	22498468	977	0.654	-3.929	8.54E-05	G/A	0.217	0.293	
rs2512276	11	124115370	975	0.692	-3.918	8.92E-05	C/G	0.406	0.494	
rs2106261	16	73051620	977	0.616	-3.912	9.15E-05	G/A	0.140	0.206	ZFHX3
rs4669131	2	7232478	977	0.697	-3.902	9.56E-05	A/G	0.374	0.464	
rs231983	3	172236440	977	0.689	-3.900	9.63E-05	A/C	0.334	0.421	
rs6825176	4	150990695	977	0.696	-3.896	9.77E-05	A/G	0.443	0.531	

A primary SNP, the missense variant rs1230345 in the *WNT1-inducible-signaling pathway protein 3* (WISP3) gene at 6q21 had the smallest p-value (n = 977, OR = 1.64, β = 4.67, p = 3.05*10^−6^). A second SNP was an intronic variant in the *Calcium Channel*, *Voltage-Dependent*, *Alpha 2/Delta Subunit 3* (CACNA2D3) gene, rs9311525. (n = 976, OR = 0.66, β = -4.41, p = 1.03*10^−5^). The last SNP found was in non-coding RNA at chromosome 7, rs1558557 (n = 977, OR = 0.67, β = -4.116, p = 3.85*10^−05^).

On the X-chromosome, a single SNP (rs41503949), an intergenic variant *near Patatin-Like Phospholipase Domain Containing 4* (PNPLA4) reached a p-value below 10^−4^ (β = 0.68, SE = 0.17, p>5.56*10^−6^). When only males were concerned, the p value kept below nominal significance (β = 0.89, SE = 0.23, p>0.0001), but not when only females were considered (β = 0.28, SE = 0.299, p = 0.34).

Additionally, SNPs investigated in previous GWAS and present on the current chip are reported in Table 2. Only five of these SNPs reached nominal significance: an intronic variant in the *vaccinia related kinase 2* (VRK2) gene, rs2312147 on chromosome 2.[48] A second SNP was also intronic in *Neurogenic locus notch homolog 4* (NOTCH4) on chromosome 6 (rs2071286). A single intronic SNP, rs7914558 on chromosome 10 in *cyclin M2* (CNNM2) also reached nominal significance. On chromosome 10, 2 SNPs reached nominal significance. The first one is the intergenic rs1602565, and finally rs12807809 near *neurogranin* (NRGN).

**Table 2:** Replication of previously reported autosomal SNPs associated with schizophrenia in GWAS studies.[9–22]. p-values were obtained using logistic regression with the first 3 principal components as covariates.

SNP	CHR	BP	n	OR	β	p	major/minor	Freq_Cases_	Freq_Controls_	Gene
rs4846033	1	11788564	977	1.058	0.121	0.904	G/A	0.010	0.009	
rs1625579	1	98502934	976	0.870	-1.162	0.245	A/C	0.164	0.184	MIR137
rs10911902	1	186632317	977	0.952	-0.424	0.672	G/A	0.183	0.191	
rs2312147	2	58222928	977	0.828	-2.037	**0.042**	G/A	0.361	0.407	VRK2
rs1344706	2	185778428	976	0.924	-0.859	0.391	A/C	0.410	0.429	ZNF804A
rs17662626	2	193984621	974	0.910	-0.571	0.568	A/G	0.086	0.094	
rs10520163	4	170626552	977	1.023	0.253	0.801	A/G	0.501	0.496	CLCN3
rs13194053	6	27143883	977	0.988	-0.092	0.927	A/G	0.165	0.174	
rs6932590	6	27248931	976	1.116	1.019	0.308	A/G	0.269	0.257	
rs928824	6	30224889	977	1.060	0.329	0.742	G/A	0.074	0.068	HCG17
rs2071286	6	32179896	977	0.780	-2.100	**0.036**	G/A	0.237	0.267	NOTCH4
rs10503253	8	4180844	977	0.843	-1.514	0.130	C/A	0.192	0.219	CSMD1
rs1155204	8	13334842	977	0.994	-0.037	0.970	A/G	0.089	0.091	DLC1
rs7004633	8	89760311	976	1.143	1.137	0.255	A/G	0.190	0.171	
rs7914558	10	104775908	976	0.803	-2.410	**0.016**	G/A	0.387	0.440	CNNM2
rs11191580	10	104906211	977	0.833	-1.069	0.285	A/G	0.075	0.087	NT5C2
rs1602565	11	29162136	977	1.355	2.155	**0.031**	A/G	0.137	0.105	
rs12807809	11	124606285	977	0.771	-2.144	**0.032**	A/G	0.156	0.193	
rs548181	11	125461709	977	0.848	-1.145	0.252	G/A	0.096	0.114	STT3A
rs1006737	12	2345295	977	1.123	1.194	0.232	G/A	0.333	0.307	CACNA1C
rs11064768	12	119818509	977	0.770	-1.726	0.084	A/G	0.088	0.111	CCDC60
rs7336332	13	28058404	977	0.975	-0.206	0.837	A/G	0.150	0.156	
rs915071	14	32433858	977	0.960	-0.453	0.651	A/G	0.486	0.496	
rs8042374	15	78908032	977	0.914	-0.821	0.412	A/G	0.222	0.238	CHRNA3
rs7192086	16	13061611	975	1.106	0.927	0.354	T/A	0.256	0.237	SHISA9
rs12966547	18	52752017	977	1.038	0.397	0.692	G/A	0.403	0.395	
rs17512836	18	53194961	977	1.041	0.158	0.875	A/G	0.034	0.033	TCF4

### Combination of rare and common variation

When analysing the combined effect of common and rare variation using the CommonRare function implemented in SKAT, one gene resulted in a p value below 10^−5^. Using one common SNP and 2 rare SNPs in WISP3 on chromosome 6, a p-value of 4.34*10^−6^ was obtained. The second best p-value is obtained by the *tumor necrosis factor (ligand) superfamily*, *member 10 gene* (TNFSF10, p = 3.49*10^-5^), followed by CACNA2D3 (p = 1.29*10^−4^). The top 5 genes with at least 3 SNPs contributing to the results are displayed in Table 3.

**Table 3:** Top 5 genes with at least 3 SNPs per gene from the SKAT CommonRare analysis, using the first 3 principal components as covariates.

Gene	CHR	BP	p	n_total_	n_test_	n_rare_	n_common_
WISP3	6	112375275–112392171	4.719E-06	3	3	2	1
TNFSF10	3	172223298–172241297	3.492E-05	3	3	2	1
CACNA2D3	5	54908632–54935282	1.285E-04	10	10	8	2
EBLN1	10	22497743–22498950	2.253E-04	3	3	1	2
CD97	19	14491313–14519537	2.283E-04	5	5	4	1

## Discussion

The current study evaluated exonic variation in a group of patients with schizophrenia and schizoaffective disorder. No SNP reached genome-wide significance levels (p< 10^−8^). At the level of genes, no gene reached genome-wide significance. These results are comparable to those of the Swedish Schizophrenia Cohort, who were also unable to find genome-wide significant results using the HumanExome Beadchip in 13000 individuals.[49] Although none of the currently investigated SNPs reached genome-wide significance, several SNPs obtained low p-values, which, combined with data from previous research, warrants further investigation.

### WISP3

The rs1230345 in the WISP3 gene had the smallest p-value of all SNPs tested. As a gene, WISP3 also had the smallest p-value from a combination of one common and 2 rare common SNPs. The WISP3 gene lies within the 6q21 region, within a larger region on chromosome 6 previously associated with schizophrenia or bipolar disorder.[50–54] Although neither WISP3 nor the neighbouring TUBE1 or LAMA4 genes, have been associated with schizophrenia, the more distant FYN gene was.[55]

The WISP3 gene belongs to the CCN family of extracellular matrix associated signalling proteins. It is mainly known for its contribution to progressive pseudo-rheumatoid dysplasia and poly-articular juvenile idiopathic arthritis. It has been linked to the intracellular accumulation of reactive oxygen species in connective tissues.[56]

Animal models have not highlighted further evidence for WISP3 as a schizophrenia candidate gene: Although WISP3 is expressed in the developing midbrain of zebrafish,[57] altering the expression of WISP3 does not affect the phenotype of mice.[58] No association of WISP3 with schizophrenia has so far been described in the current literature. Further research is needed to confirm the possible role of this gene or variant in schizophrenia.

### CACNA2D3

The CACNA2D3 gene forms a subunit of the L-type gated calcium channel, where it influences the trafficking and kinetic or voltage-dependent properties.[59] CACNA2D3 lies within the 3p14.3 region. The 3p14 region has been associated with schizophrenia in a single study,[60] and another study found an association the 3p14 region and the antisaccade endophenotype in schizophrenia.[61]

As one of the regulators in the calcium pathway, CACNA2D3 is an interesting candidate gene for schizophrenia as the calcium pathway is thought to be a major contributor to the genetic risk of schizophrenia or bipolar disorder,[62–64] with several studies linking genes of this pathway to both disorders.[19,21,22,65–67]

Several studies of the CACNA2D3 gene reported associations with symptoms of schizophrenia. The CACNA2D3 gene has been shown to alter pain sensitivity in both animals and humans.[68] Patients with schizophrenia display a diminished pain sensitivity, as was shown in a meta-analysis of experimental studies, independent of treatment status.[69] Knockout mice for CACNA2D3 have a decreased startle reflex. [70] The startle reflex modulation, as measured by prepulse inhibition, is a putative endophenotypes of schizophrenia.[71] In an exome sequencing study of autism, a single subject suffering from autism also had a mutation in CACNA2D3.[72] Given the evidence for members of the calcium pathway in schizophrenia, this variant could be of interest for further research.

### TNFSF10

Although no single SNP emerged from the TNFSF10 gene, a joint analysis of rare and common variants resulted in a gene with the second lowest gene-wide p-value. The TNFSF10 gene plays a role in the p53-mediated programmed cell death, which is activated after cells get exposed to DNA damage.[73] Previous research has implicated modulations in cell apoptosis in schizophrenia,[74,75] but no direct link between the current gene, apoptosis and schizophrenia was found.

Only a single reference of this gene was found in connection with schizophrenia. In a study based on the dataset of the Stanley Neuropathology Consortium, comparing gene expression in bipolar disorder and schizophrenia versus controls, TNFSF10 had a significant contribution to the support vector machine algorithm for classification of schizophrenia or bipolar versus controls.[76].

### Replication of previous findings

Table 2 contains the results of SNPs previously associated with schizophrenia in large GWAS studies.

### Limitations

The current study has a moderate sample size. This disadvantage is partially offset by using samples with clear diagnosis of schizophrenia and schizoaffective disorder, and having an ethnically homogeneous sample. DNA was of high quality, and quality control resulted in 99.8% of designed SNVs available for analysis.

## Conclusion

The current investigation of 493 patients with schizophrenia or schizoaffective disorders versus 484 healthy controls did not reveal any variant with genome-wide significant p-values. Amongst the lowest p-values were 2 genes that might be of theoretical interest: CACNA2D3, directly involved in regulating the intracerebral calcium homeostasis, and TNFSF10, a gene that is involved in apoptosis in schizophrenia. However, given the limited sample size and thus limited power, these results are preliminary at best and should be treated with caution.

## Supporting Information

S1 Data The raw_data.zip file contains the tplink files of the current analysis, after all quality checks were done. (DOCX)

S1 Fig MDS plot of ethnicity compared with 1000 Genomes. Comparison of ethnicity of the current sample with the 1000 genomes August 2010 release. Study subjects are colored black (code OUR), and lie within the Caucasian cluster together with the Utah residents (CEU), British subjects (GBR) and Italians (TSI). The upper right cluster is formed by Americans of African descent (ASW), Puerto Rica (PUR) and Nigerians (YRI). In the lower cluster, Han Chinese (CHS and CHB) and Japanse (JPT) subjects cluster together. Lastly, Finns (FIN) are between the European and Asian clusters. Based upon this figure, 3 additional samples to the right were removed. (final n = 977).
(TIF)

S2 Fig MDS plot of ethnicity. First and second principal component generated by the MDS algorithm of KING in the current dataset.
(PNG)

S3 Fig QQ plot of the PCA corrected logistic regression. (TIF)
