# Supplementary material for: Genetic Evaluation of Schizophrenia Using the Illumina HumanExome Chip
Source: PLoS One. 2016 Mar 30;11(3):e0150464. doi: 10.1371/journal.pone.0150464 (PMC4814136; doi:10.1371/journal.pone.0150464)
Supplement: S1 Data — (DOCX) [file pone.0150464.s001.docx]

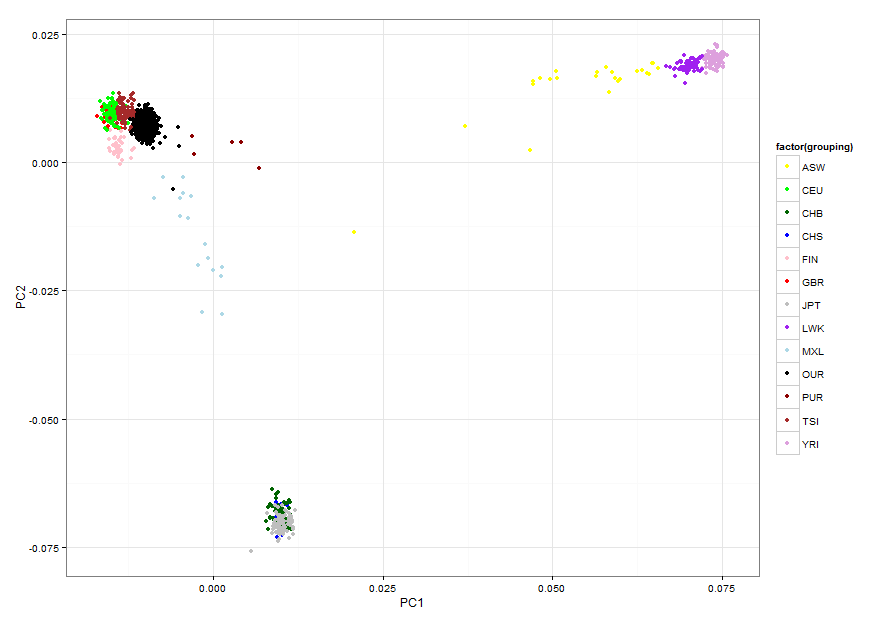


S1 Fig. MDS plot of ethnicity compared with 1000 Genomes. Comparison of ethnicity of the current sample with the 1000 genomes August 2010 release. Study subjects are colored black (code OUR), and lie within the Caucasian cluster together with the Utah residents (CEU), British subjects (GBR) and Italians (TSI). The upper right cluster is formed by Americans of African descent (ASW), Puerto Rica (PUR) and Nigerians (YRI). In the lower cluster, Han Chinese (CHS and CHB) and Japanse (JPT) subjects cluster together. Lastly, Finns (FIN) are between the European and Asian clusters. Based upon this figure, 3 additional samples to the right were removed. (final n = 977)


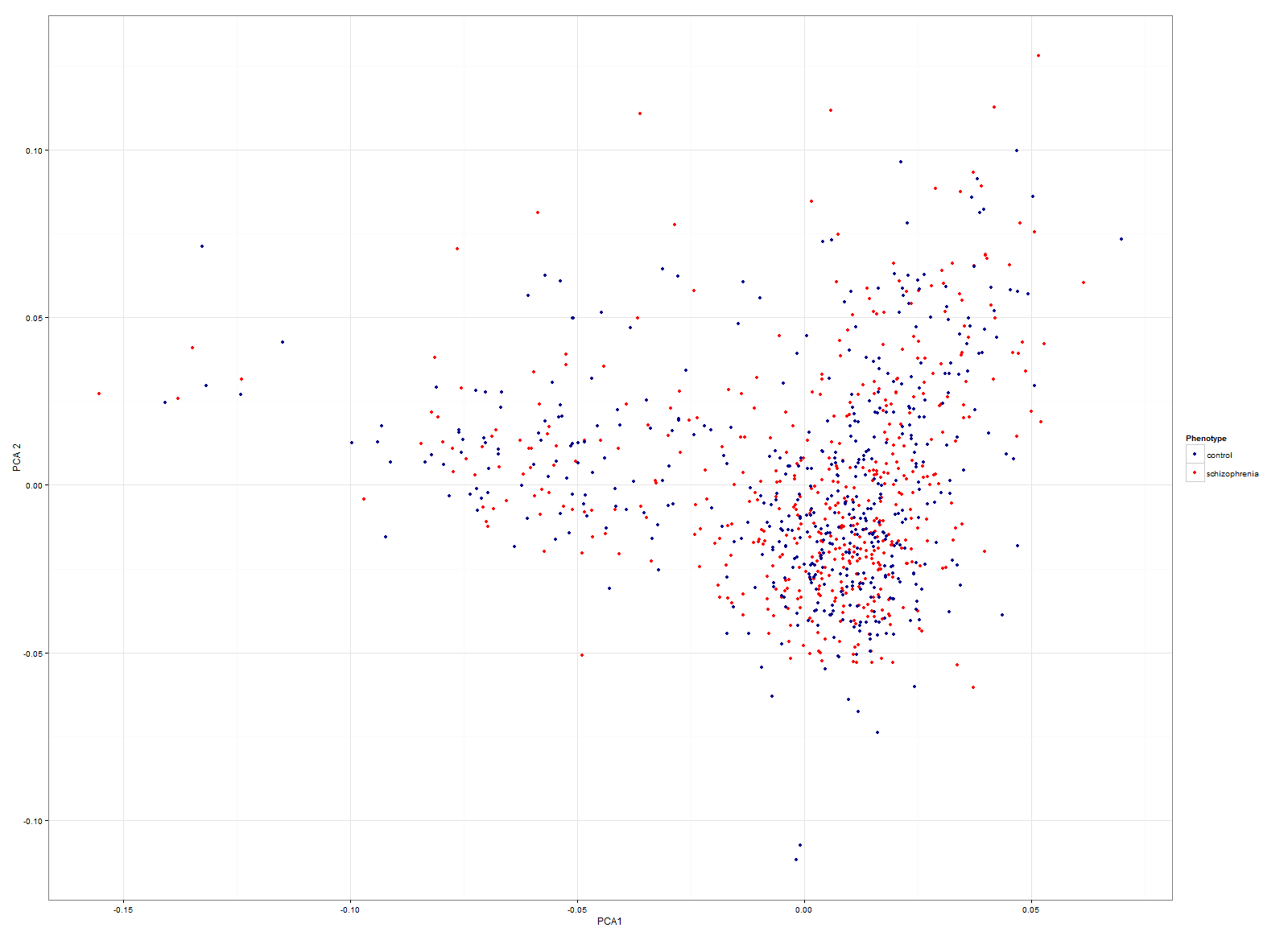


S2 Fig. MDS plot of ethnicity. First and second principal component generated by the MDS algorithm of KING in the current dataset.


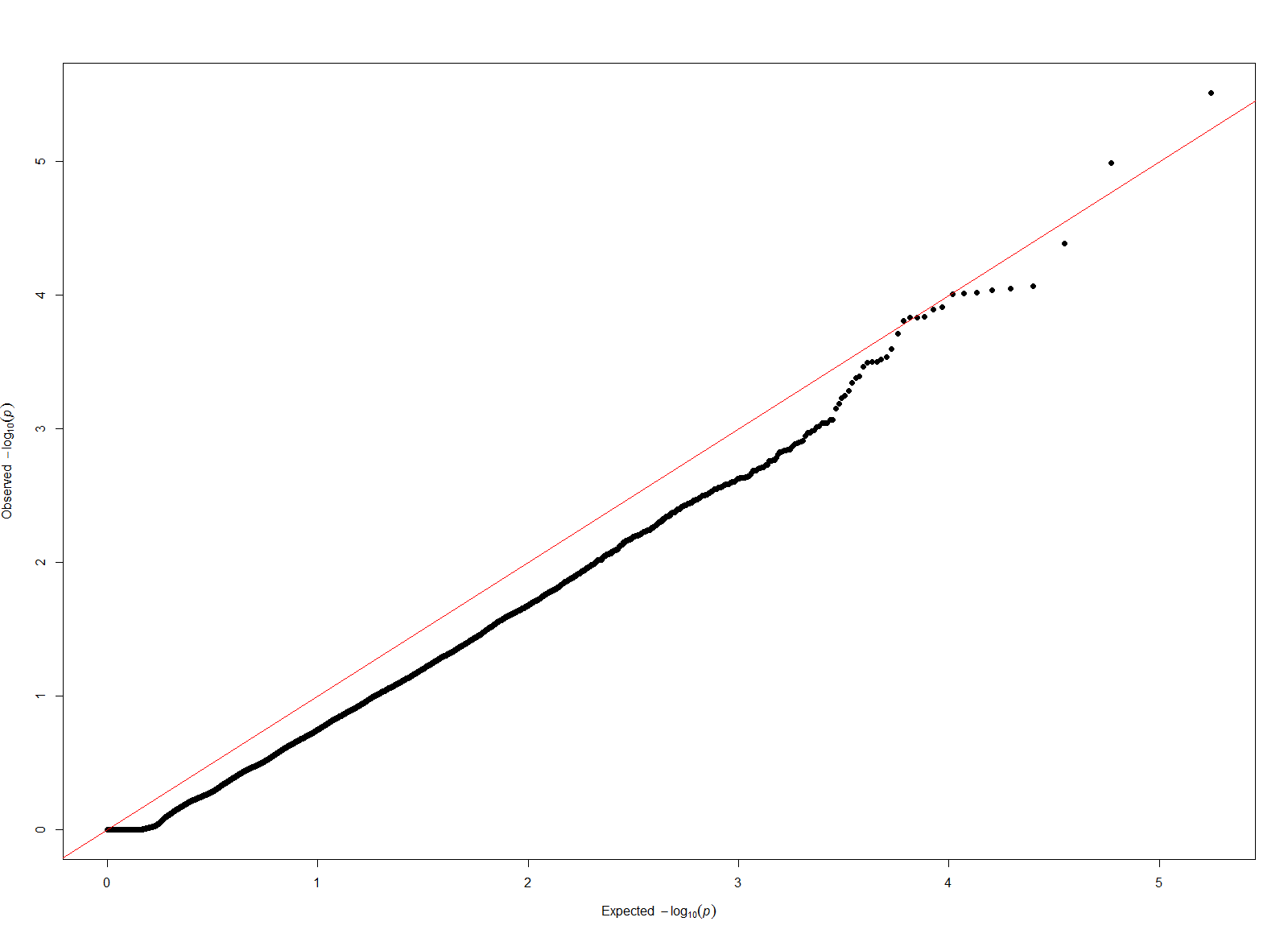


S3 Fig. QQ plot of the PCA corrected logistic regression.
